# Assessment of Free Radical Scavenging Activity of Dimethylglycine Sodium Salt and Its Role in Providing Protection against Lipopolysaccharide-Induced Oxidative Stress in Mice

**DOI:** 10.1371/journal.pone.0155393

**Published:** 2016-05-12

**Authors:** Kaiwen Bai, Wen Xu, Jingfei Zhang, Tao Kou, Yu Niu, Xiaoli Wan, Lili Zhang, Chao Wang, Tian Wang

**Affiliations:** College of Animal Science and Technology, Nanjing Agricultural University, No. 6, Tongwei Road, Xuanwu District, Nanjing 210095, People's Republic of China; National Institutes of Health, UNITED STATES

## Abstract

In the present study, the free radical scavenging activities (against 1,1-diphenyl-2-pierylhydrazy (DPPH), 2,2'-Azinobis-(3-ethylbenzthiazoline-6- sulphonate) (ABTS^+^), Hydrogen peroxide (H_2_O_2_)) of dimethylglycine sodium salt (DMG-Na) were measured and compared with those of Trolox (6-hydroxy-2, 5, 7, 8-tetramethylchroman-2-carboxylic acid), a commonly used antioxidant. The radical scavenging activities of DMG-Na were found to be the highest at 40 mg/ml. In *Experiment 2*, gastric intubation in mice with 12 mg DMG-Na/0.3 ml sterile saline solution significantly increased (*P* < 0.05) the body weight (BW) (28 d), organ proportion (liver and spleen), and antioxidant capacity in serum and the liver (Superoxide dismutase (SOD), Hydrogen peroxidase (CAT), Glutathione peroxidase (GPx), and Total antioxidant capacity (T-AOC)), and significantly decreased (*P* < 0.05) the activities of serum Glutamic-pyruvic transaminase (ALT) and Glutamic oxalacetic transaminase (AST) and Methane Dicarboxylic Aldehyde (MDA) contents in the serum and liver. Specifically, the effect of 12 mg DMG-Na/0.3 ml sterile saline solution, which showed the highest antioxidant capacity, was further studied using a mice model. In *Experiment 3*, the mice CL (CON+ lipopolysaccharide (LPS)) group showed a significant decrease (*P* < 0.05) in the serum ALT and AST content; hepatic mitochondrial antioxidant capacity (Manganese Superoxide dismutase (MnSOD), Glutathione reductase (GR), GPx, Glutathione (GSH)); MDA and Protein carbonyl (PC) content; Reactive oxygen species (ROS) level, Mitochondrial membrane potential (MMP) level, and expression of liver antioxidant genes (Nuclear factor erythroid 2-related factor 2 (*Nrf2*), Heme oxygenase 1 (*HO-1*), Manganese superoxide dismutase (*MnSOD*), Glutathione peroxidase 1 (*Gpx1*), Sirtuin 1 (*Sirt1*)) relative to the mice CS (CON+ sterile saline) group. The DL (DMG+LPS) group showed a significant decrease (*P* < 0.05) in serum ALT and AST content, ROS level, and expression of liver antioxidant gene *MnSOD*, *Gpx1*, *Sirt1* and a significant increase (*P* < 0.05) in the hepatic mitochondrial antioxidant capacity (MnSOD, GSH, GPx, GR) and MMP level relative to the CL group. These results indicate that DMG-Na could protect against the LPS-induced oxidative stress by enhancing the free radical scavenging capacity, and increasing the activity of antioxidant defense system.

## Introduction

Lipid peroxidation, a process contributing to the development of oxygen radical-related damages, is one of the major causes of cell membranes damages [1]. Oxidative stress is induced when the balance between the antioxidant defense system and free radical generation system is disturbed, leading to several diseases. Previous studies have indicated that generation of free radicals is probably one of the mechanisms leading to diseases [2], including cancer [3], and neuronal disorders [4]. LPS is a cell wall component of gram-negative bacteria, and can cause severe inflammation, septic shock, and systemic inflammatory response syndrome [5]. Oxidative stress induced by LPS involves free radicals, such as ROS and reactive nitrogen species (RNS) [6]. Wichterman et al. [7] suggested that LPS from gram-negative bacteria could be very useful in studying oxidative stress in laboratory animals. Cadenas et al. [8] found that LPS promotes the generation of free radicals by altering the activity of the major physiological sources of free radicals in the mitochondria. LPS also induces oxidative stress in the liver [9], and produces typical hepatic injury [10]. There is a complex system of natural enzymatic and non-enzymatic antioxidants in human body that defends against oxidative stress caused by free radicals and oxidative materials. Recent studies suggest that dietary supplementation of antioxidants is beneficial in preventing diseases and for improving the quality of life. These antioxidants act by reducing the free radicals. Antioxidant enzymes or natural products can reduce oxidative stress by antioxidation.

**Fig 1:**
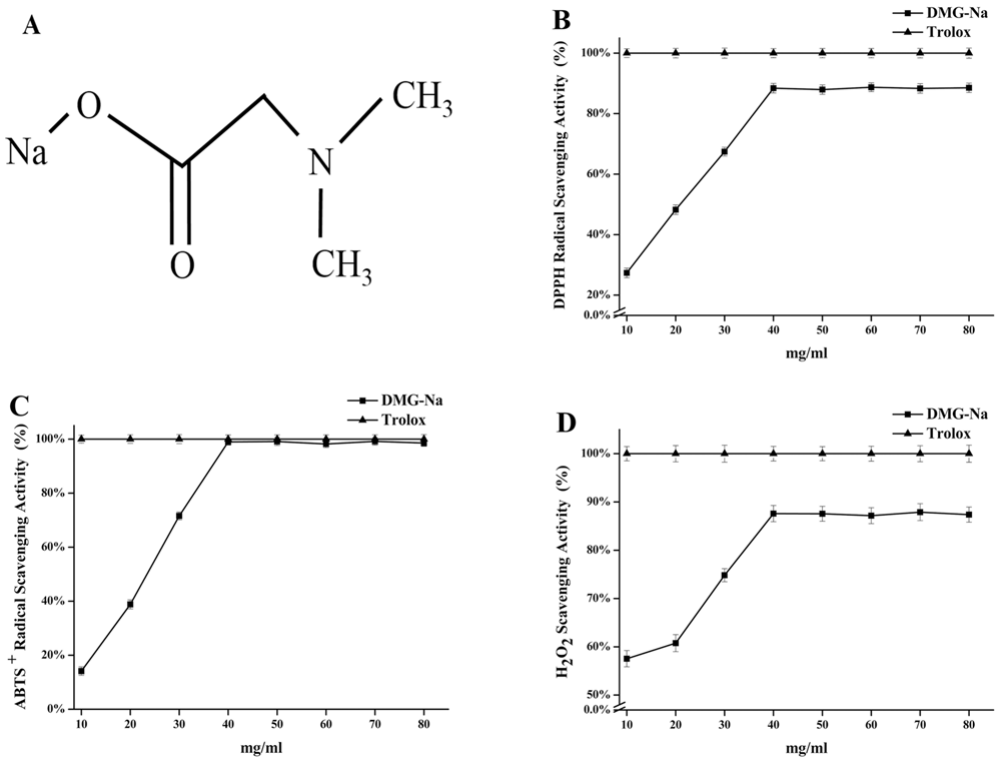
The structures of DMG-Na (Fig 1A), DPPH (Fig 1B), ABTS ^+^ (Fig 1C), and H_2_O_2_ (Fig 1D). Radical scavenging effects of DMG-Na and Trolox at 10–80 mg/ml. In panels 1B, 1C, and 1D, the values of the Trolox group is set to 100%. Values of DPPH and ABTS^+^ radical scavenging ability, and H_2_O_2_ scavenging ability are expressed as the mean ± SEM of three independent experiments. 1,1-diphenyl-2-pierylhydrazy (DPPH); 2,2'-Azinobis-(3-ethylbenzthiazoline-6- sulphonate) (ABTS^+^).

N, N-Dimethylglycine (DMG) is a lesser-known substance and is related to glycine metabolism (Fig 1A). It is similar to choline and betaine, and acts as their intermediary metabolite in the body [11]. Friesen et al. [12] found that DMG could act as a source of glycine for glutathione synthesis, and thereby, may improve the antioxidant capacity in the body. Other researchers suggested that dietary supplementation with DMG could reduce oxidative stress and improve athletic performance in men [13], dogs [14], and horses [15]. Cupp et al. [16] reported that DMG could be absorbed rapidly and completely through gastric intubation in mice, generally used for the uptake of small water-soluble molecules. Clapés et al. [17] suggested that the effect of DMG-Na on growth performance was owing to the surfactant properties of the esters. Kalmar et al. [18] also found that dietary supplementation with 0 to 1 g/kg DMG-Na could improve body performance. Moreover, DMG, acting as a methyl donor, could improve immunity, function as an antioxidant to prevent oxidative stress, and scavenge excess of free radicals to avoid unwanted reactions in the body [19]. Levine et al. [20] reported that DMG not only improved the utilization of oxygen to protect the body from excess of free radical induced oxidative stress, but also enhanced the immune response of individuals. The study was designed to measure the free radical scavenging activity of DMG-Na and its protective effects against the oxidative stress induced by LPS in mice.

## Materials and Methods

### Experiment 1: Free radicals scavenging activity of DMG-Na *in vitro*

#### DPPH radical scavenging activity

DPPH radical scavenging activity was estimated following the method of [21] with some changes. DPPH was mixed with ethanol to get a 0.1 mM solution and stored in dark. Subsequently, Trolox or DMG-Na was mixed at concentrations ranging from 10 to 80 mg/ml and shaken vigorously. The solution was then incubated in dark at 24°C for 30 min before measuring the absorbance at 517 nm, with a spectrophotometer. DPPH radical scavenging activity was calculated using the equation below:

$$DPPH\text{ radical scavenging activity }\left(\%\right)=\frac{{\text{A}}_{\text{control}}-{\text{A}}_{\text{sample }}}{{\text{A}}_{\text{control}}}*100\%$$

where, A_control_ was the absorbance of the control and A_sample_ was the absorbance of the sample under the same conditions.

#### ABTS^+^ radical scavenging activity

We followed the method of Siddhuraju P and Manian S [22], in which the reaction was based on the color changes. ABTS^+^ working solution was formed by mixing 7 mM ABTS^+^ stock solution and 2.45 mM K_2_S_2_O_8_ solution and then incubated in dark at 24°C for 12–16 h. ABTS^+^ solution was diluted with ethanol before use to get an absorbance of 0.70 ± 0.02 at 734 nm. One milliliter ABTS^+^ solution was incubated at 30°C with 3 ml Trolox or DMG-Na solution at concentrations ranging from 10 to 80 mg/ml for 30 min before measuring the absorbance at 534 nm. ABTS^+^ radical scavenging activity was calculated using the equation below:

$$ABTS{\;}^{+}\text{radical scavenging activity }\left(\%\right)=\frac{{\text{A}}_{\text{control}}-{\text{A}}_{\text{sample }}}{{\text{A}}_{\text{control}}}*100\%$$

where, A_control_ was the absorbance of the control and A_sample_ was the absorbance of the sample under the same conditions.

#### H_2_O_2_ scavenging activity

H_2_O_2_ scavenging assay was performed by the method of Zhang [23]. Briefly, 3.4 ml of Trolox or DMG-Na solution at concentrations ranging from 10 to 80 mg/ml in phosphate buffer (0.1 M, pH = 7.4) were mixed with 0.6 ml of 43 mM H_2_O_2_ solution before the absorbance was recorded at 230 nm. H_2_O_2_ radical scavenging activity was calculated using the following equation:

$$H_{2}O_{2}\text{scavenging activity}\;\left(\%\right)\;=\;\frac{{\text{A}}_{\text{control}}-{\text{A}}_{\text{sample }}}{{\text{A}}_{\text{control}}}*100\%$$

where, A_control_ was the absorbance of the control and A_sample_ was the absorbance of the sample under the same conditions.

### Experiment 2: Effects of DMG-Na on antioxidant capacity of mice

#### Materials

Forty male Kunming mice with BW in the range of 20–25 g were obtained from the Animal Multiplication Centre of Qinglong Mountain. The mice were randomly assigned to four treatment groups, with ten mice in each group. D-1, D-2, and D-3 groups were with 9, 12, and 15 mg DMG-Na, which was dissolved with 0.3 ml of sterile saline solution respectively, and administered to rats via gastric intubation for 28 days, and each mouse was given 0.3 ml dose twice daily. The control group was given sterile saline solution at the same volume. All the mice were fed common basal diets. The mice were sedated during intubation and no mortality occurred. DMG-Na was procured from Qilu Sheng Hua Pharmaceutical Co., Ltd., Shandong, People's Republic of China.

#### Husbandry

Four groups of mice were raised under controlled conditions with 25 ± 3°C temperature, 60 ± 10% humidity, and a 12/12 light-dark cycle. The mice were provided water and diet *ad libitum*. The experiment was approved and conducted under the supervision of Animal Care and Use Committee, Nanjing Agriculture University, Nanjing, People's Republic of China, and adopted the Animal Care and Use Guidelines for all the animals used in this experimental procedures. In this study, all efforts are taken to minimize suffering when the mice meets our euthanasia criteria. Progressive deterioration of the animals' health leading to death is not allowed. The humane endpoint is set to decide when to sacrifice the mice, which includes that body temperature and physical activity are significantly worse than the active mice and are decreased or not increase in a few hours, the mice are no response to intermittent stimulation 3 times in half an hour, or the respiratory rate of mice are rapidly or slowly apparently. The mice used in this study were taken care by trained workers in Nanjing Agriculture University, Nanjing, People's Republic of China. They monitored the health of each mice every 6 h and strictly performed the rules of humane endpoints to determine when the mice should be euthanized. At 29 day of raising, all the mice were anesthetized by intraperitoneal injection of 100 mg/kg pentobarbital (Sigma, USA) and sacrificed under the condition of limb paralysis or unable to right themselves in 15 seconds when placed on their side.

#### Measurement of serum ALT and AST

All the mice were anesthetized and sacrificed after 28 days of gastric intubation with DMG-Na. The blood was collected and centrifuged at 3500 rpm at 4°C for 15 min, and the serum ALT and AST levels were measured using corresponding diagnostic kits (Nanjing Jiancheng Bioengineering Institute, Nanjing, P. R. China) according to the manufacturers’ instructions.

#### Determination of antioxidant system

After 28 days of gastric intubation with DMG-Na, the 40 selected male mice were anesthetized and sacrificed, and their blood and liver were collected. One gram mice liver was homogenized at 8000 rpm for 10 s in 9 ml of 0.9% sodium chloride buffer on ice and centrifuged at 4000 rpm at 4°C for 15 min. The blood was centrifuged at 3500 rpm at 4°C for 15 min. Supernatant of the liver homogenization solution and serum were individually used to measure the activities of SOD, CAT, GPx, and T-AOC using corresponding diagnostic kits (Nanjing Jiancheng Bioengineering Institute, Nanjing, P. R. China) according to the instructions of the manufacturer.

#### Measurement of lipid peroxidation

Lipid peroxidation, expressed as malondialdehyde concentration, was determined using a MDA assay kit (Nanjing Jiancheng Bioengineering Institute, Nanjing, P. R. China) according to the instructions of the manufacturer. Briefly, liver homogenates (0.9% sodium chloride buffer to produce a 10% tissue lysate) were used to calculate liver MDA levels by the method of thiobarbituric acid (TBA). The MDA-TBA mixture produced during the reaction of MDA in samples with TBA was measured at 535 nm (UV-2401PC, Shimadzu, Japan).

### Experiment 3: Protective effects of DMG-Na against LPS-induced oxidative stress in mice

#### Materials

Forty male Kunming mice with a BW of 20–25 g were obtained from the Animal Multiplication Centre of Qinglong Mountain. All the mice were randomly assigned to four treatment groups with each treatment having ten mice. DMG groups (DS, DL) were with 12 mg DMG-Na, which was dissolved with 0.3 ml of sterile saline solution respectively and administered to rats via gastric intubation for 28 days, and each mouse was given 0.3 ml dose twice daily. The control groups (CS, CL) were treated with sterile saline solution at the same volume. All the mice were fed common basal diets. The mice were sedated during intubation and no mortality occurred. LPS (*Escherichia coli*, 0111:B4, purchased from Sigma, USA) was prepared in 0.9% sterile saline solution. At 29 day, mice of DL and CL group were intraperitoneal injection with 100 μg/kg body weight of LPS, whereas the mice of DS and CS group were intraperitoneal injection with 100 μg/kg body weight of 0.9% sterile saline solution.

#### Husbandry

The husbandry was done according to the method described in *Experiment 2*.

#### Determination of serum ALT and AST

Forty selected male mice were anesthetized and slaughtered 24 h after the LPS injection. Blood was obtained from the sacrificed animals and centrifuged at 3500 rpm for 15 min at 4°C. The measurement of ALT and AST in serum are described in *Experiment 2*.

#### Isolation of mice liver mitochondria

Hepatic mitochondria were prepared according to the method described by Tang [24]. Namely, liver tissue was homogenized in ice-chilled Dounce homogenizers (1:10, w/v) using isolation buffer containing 10 mM MOPS pH 7.4, 250 mM sucrose, 5 mM KH_2_PO_4_, 2 mM MgCl_2_, 1 mM EGTA, and 0.1% fatty acid-free BSA, and centrifuged at 1,000 g for 5 min at 4°C. Remove the supernatants and resuspend the mitochondria-enriched pellets gently, and washed with the isolation buffer, then obtained the pelleted by centrifugation at 12,000 g for 5 min. Mitochondria were lysed and the protein was measured using the Micro BCA protein assay kit (Nanjing Jiancheng Bioengineering Institute, Nanjing, P. R. China) according to the manufacturers’ instructions.

#### Detection of mitochondria antioxidant system

Concentrations of protein in the mitochondria and activities of MnSOD, GR, GPx, and GSH in the mitochondria of mice liver were measured using corresponding diagnostic kits (Nanjing Jiancheng Bioengineering Institute, Nanjing, P. R. China) according to the instructions of the manufacturer.

#### Measurement of lipid peroxidation and protein oxidation

Lipid peroxidation in mice liver mitochondria was determined according to the method described in *Experiment 2*. Protein oxidation of mice liver mitochondria was calculated using the concentrations of the PC. The PC concentration was measured by using a previously described method [25] and presented in nmol/mg protein.

#### Determination of ROS

ROS level in mice liver were detected using a ROS assay kit (Nanjing Jiancheng Bioengineering Institute, Nanjing, *P*. *R*. China) according to the manufacturers’ instructions. Briefly, the mitochondrial was incubated with 2’,7’-Dichlorofluoreseindiacetate (DCFH-DA) (10 μM) and DNA stain Hoechst 33342 (10 mmol/L) at 37°C for 30 min. Then the DCFH fluorescence of the mitochondrial was measured at an emission wavelength of 530 nm and an excitation wavelength of 485 nm with a FLX 800 microplate fluorescence reader (Biotech Instruments Inc., USA). The results were expressed as the mean DCFH-DA fluorescence intensity over that of the control.

#### Measurement of mitochondrial membrane potential

The changes of MMP level in mice liver was detected using the MMP assay kit (Beyotime Institute of Biotechnology, Jiangsu, China) according to the instructions of the manufacturer. Namely, the mitochondrial were loaded with 1×JC-1 at 37°C for 20 min, then washed and analyzed by flow cytometry (FACS Aria III, BD, New Jersey, US). MMP level can be calculated as an increasing green fluorescent/red fluorescent intensity ratio. When MMP levels are low, JC-1 exists mainly as a monomer, which emits green fluorescence (excitation wavelength of 490 nm and emission wavelength of 540 nm). However, when MMP levels are high, JC-1 exists mainly as a polymer, which emits red fluorescence (excitation wavelength of 525 nm and emission wavelength of 590 nm). The results were calculated as the fluorescence ratio of aggregates (red) to monomers (green).

#### Quantitative real-time PCR analyses

Total RNA was obtained from the mice liver using Trizol Reagent (TaKaRa, Dalian, China) and then reverse-transcribed using a commercial kit (Perfect Real Time, SYBR^®^ PrimeScript^™^ TaKaRa, China) following the instructions of the manufacturer. The mRNA expression levels of specific genes were quantified via real-time PCR, using SYBR^®^ *Premix Ex Taq*^™^ II (Tli RNaseH Plus) and an ABI 7300 Fast Real-Time PCR detection system (Applied Biosystems, USA). The SYBR Green PCR reaction mixture consisted of 10 μl SYBR^®^ *Premix Ex Taq* (2X), 0.4 μl of the forward and reverse primers, 0.4 μl of ROX reference dye (50X), 6.8 μl of ddH_2_O and 2 μl of cDNA template. Each sample was amplified in triplicate. The fold-expression of each gene was calculated according to the 2^-**ΔΔ**Ct^ method [26], in which the *β-Actin* gene was used as an internal standard. The primer sequences used are given in Table 1.

**Table 1:** Primer sequences used for Real-time PCR assay.

Name ^1^	Sequence (5’→3’) ^2^	Genbank ^3^
***β-Actin***	CTGTCCCTGTATGCCTCTG	NM_007393.3
	ATGTCACGCACGATTTCC	
***Nrf2***	CAGTGCTCCTATGCGTGAA	NM_010902.3
	GCGGCTTGAATGTTTGTC	
***HO-1***	ACAGATGGCGTCACTTCG	NM_010442.2
	TGAGGACCCACTGGAGGA	
***MnSOD***	CCGAGGAGAAGTACCACGAG	NM_013671.3
	GCTTGATAGCCTCCAGCAAC	
***GPx1***	AGTATGTGTGCTGCTCGGCTCT	NM_008160.6
	CCAGTAATCACCAAGCCAATGC	
***NQO1***	CTTTAGGGTCGTCTTGGC	NM_008706.5
	CAATCAGGGCTCTTCTCG	
***SIRT1***	TGCAGACGTGGTAATGTCCAAAC	NM_019812.2
	ACATCTTGGCAGTATTTGTGGTGAA	

^1^ Huclear factor erythroid 2-related factor 2 (*Nrf2*); Heme oxygenase 1 (*HO-1*); Manganese superoxide dismutase (*MnSOD*); Glutathione peroxidase 1 (*Gpx1*); NAD(P)H quinone oxidoreductase 1 (*NQO1*); Sirtuin 1 (*Sirt1*).

^2^ Shown as forward primer followed by reverse primer.

^3^ GenBank Accession Number.

### Statistical analysis

All the data were subjected to one-way ANOVA using the GLM procedure of the Statistical Analysis System (SAS Institute Inc., Cary, NC). When the F test was significant, means were compared using one-factor ANOVA and Bonferroni’s multiple-comparisons test. The differences between the treatment groups were considered significant at *P* < 0.05. Results are presented as means ± SEM.

## Results

### Experiment 1: Free radicals scavenging activity of DMG-Na *in vitro*

The DPPH radical scavenging capacity of DMG-Na in comparison to that of the synthetic antioxidant Trolox is shown in Fig 1B. At concentrations from 10 to 80 mg/ml, the DPPH radical scavenging capacity of Trolox is set to 100%. At 40 mg/ml, DMG-Na showed the highest (88.40%) DPPH radical scavenging activity compared with Trolox.

At 10 to 80 mg/ml concentration, the ABTS^+^ radical scavenging capacity of Trolox is set to 100% (Fig 1C). DMG-Na achieved the highest (98.90%) ABTS^+^ radical scavenging capacity compared with Trolox at 40 mg/ml.

Fig 1D shows the H_2_O_2_ scavenging activity of DMG-Na and Trolox at concentrations ranging from 10 to 80 mg/ml. The H_2_O_2_ scavenging capacity of Trolox is set to 100%. DMG-Na showed the highest (87.58%) H_2_O_2_ scavenging capacity compared with Trolox at 40 mg/ml.

### Experiment 2: Effects of DMG-Na on antioxidant capacity of mice

Gastric intubation with DMG-Na at 12 mg/0.3 ml sterile saline solution significantly increased (*P* < 0.05) the BW of mice at 28 days compared with the BW of CON mice (Table 2). During the 28 days, food intake was not affected by the gastric intubation of DMG-Na in mice. The organ proportion of mice after 28 days of gastric intubation is presented in Table 2. The liver and spleen proportions significantly increased (*P* < 0.05). However, no difference was observed in the pancreas and kidney proportions during the trial compared to the CON mice.

**Table 2:** Effects of DMG-Na on mice growth performance and organ proportion^1^.

	Treatment ^3^			
Item ^2^	CON	D-1	D-2	D-3
**Food Intake (g)**				
**1 – 28d**	**177.52±1.21**	**173.88±1.36**	**170.80±1.33**	**172.76±1.45**
**Growth performance (g)**				
**BW 0d**	**24.97±0.38**	**24.38±0.38**	**24.56±0.41**	**24.76±0.47**
**BW 28d**	**47.61±0.55**^c^	**48.47±0.40**^b^^c^	**51.44±0.57**^a^	**49.35±0.34**^b^
**Organ proportion (%)**				
**Liver**	**4.81±0.18**^c^	**5.10±0.11**^b^	**5.30±0.10**^a^	**5.14±0.16**^b^
**Spleen**	**0.31±0.08**^b^	**0.41±0.06**^a^	**0.45±0.08**^a^	**0.41±0.05**^a^
**Pancreas**	**0.44±0.03**	**0.45±0.05**	**0.48±0.03**	**0.47±0.03**
**Kidney**	**0.63±0.04**	**0.65±0.05**	**0.66±0.05**	**0.64±0.06**

^a-c^ Means in the same row with different superscripts differ (*P* < 0.05).

^1^ CON, gastric intubation in mice with 0 mg DMG-Na/0.3 ml sterile saline solution; D-1, gastric intubation in mice with 9 mg DMG-Na/0.3 ml sterile saline solution; D-2, gastric intubation in mice with 12 mg DMG-Na/0.3 ml sterile saline solution; D-3, gastric intubation in mice with 15 mg DMG-Na/0.3 ml sterile saline solution.

^2^ BW, Body weight (per mouse); Food Intake (per mouse).

^3^ Each mean represents one replicate pens with 10 mice per pen.

**Fig 2:**
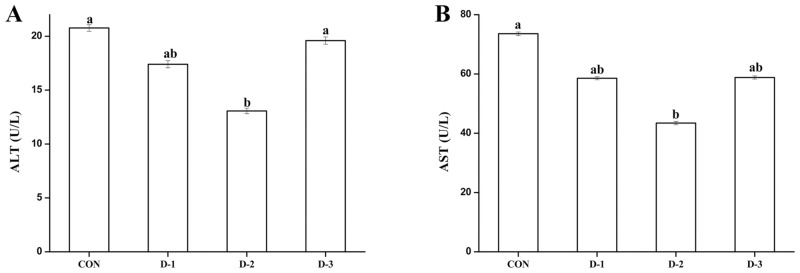
Effects of DMG-Na on ALT (A) and AST (B) activities in mice serum. Values are mean ± SEMs (n = 10). Mean of a variable without a common letter differ, *P* < 0.05. Alanine aminotransferase (ALT); Aspartate aminotransferase (AST). CON, gastric intubation in mice with 0 mg DMG-Na/0.3 ml sterile saline solution; D-1, gastric intubation in mice with 9 mg DMG-Na/0.3 ml sterile saline solution; D-2, gastric intubation in mice with 12 mg DMG-Na/0.3 ml sterile saline solution; D-3, gastric intubation in mice with 15 mg DMG-Na/0.3 ml sterile saline solution.

Gastric intubation with DMG-Na at 12 mg/0.3 ml sterile saline solution significantly lowered the ALT and AST concentrations in the mice serum (*P* < 0.05) compared to those in the mice of the CON group (Fig 2A and 2B).

**Fig 3:**
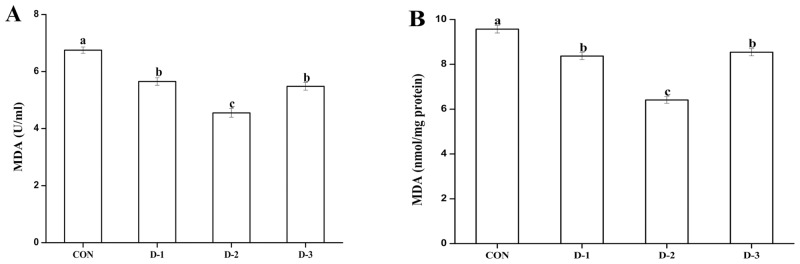
Effects of DMG-Na on MDA concentrations in mice serum (A) and liver (B). Values are mean ± SEMs (n = 10). Mean of a variable without a common letter differ, *P* < 0.05. Malondialdehyde (MDA). CON, gastric intubation in mice with 0 mg DMG-Na/0.3 ml sterile saline solution; D-1, gastric intubation in mice with 9 mg DMG-Na/0.3 ml sterile saline solution; D-2, gastric intubation in mice with 12 mg DMG-Na/0.3 ml sterile saline solution; D-3, gastric intubation in mice with 15 mg DMG-Na/0.3 ml sterile saline solution.

Compared to the CON group, gastric intubation with DMG-Na at 12 mg/0.3 ml sterile saline solution significantly improved the activities of the antioxidant system (*P* < 0.05) in both mice serum and liver (Table 3). It also significantly reduced the MDA contents (*P* < 0.05) in these organs (Fig 3A and 3B).

**Table 3:** Effects of DMG-Na on antioxidant system in mice serum and liver ^1^.

	Treatment ^3^			
Item ^2^	CON	D-1	D-2	D-3
**Serum**				
**SOD (U/ml)**	**69.72±0.53**^c^	**71.65±0.41**^b^^c^	**80.35±0.44**^a^	**75.68±0.50**^b^
**CAT (U/ml)**	**6.49±0.13**^c^	**7.34±0.11**^b^^c^	**8.16±0.15**^a^	**7.67±0.14**^b^
**GPx (U/ml)**	**72.54±0.37**	**87.65±0.55**^a^^b^	**91.89±0.46**^a^	**82.93±0.51**^b^
**T-AOC (U/ml)**	**1.67±0.05**^b^	**1.76±0.05**^a^	**1.89±0.06**^a^	**1.81±0.04**^a^
**Liver**				
**SOD (U/mg prot)**	**122.54±1.65**^c^	**172.32±1.91**^b^	**230.53±2.87**^a^	**185.34±1.88**^b^
**CAT (U/mg prot)**	**11.11±0.35**^c^	**11.80±0.41**^c^	**17.70±0.42**^a^	**15.68±0.56**^b^
**GPx (U/mg prot)**	**79.52±1.07**^c^	**98.95±1.14**^b^	**131.42±1.15**^a^	**108.86±1.25**^b^
**T-AOC (U/mg prot)**	**1.50±0.05**^c^	**1.67±0.05**^b^	**2.43±0.06**^a^	**1.65±0.05**^b^

^a-c^ Means in the same row with different superscripts differ (*P* < 0.05).

^1^ CON, gastric intubation in mice with 0 mg DMG-Na/0.3 ml sterile saline solution; D-1, gastric intubation in mice with 9 mg DMG-Na/0.3 ml sterile saline solution; D-2, gastric intubation in mice with 12 mg DMG-Na/0.3 ml sterile saline solution; D-3, gastric intubation in mice with 15 mg DMG-Na/0.3 ml sterile saline solution.

^2^ Superoxide dismutase (SOD), Hydrogen peroxidase (CAT), Glutathione peroxidase (GPx), Total antioxidant capacity (T-AOC).

^3^ Each mean represents one replicate pens with 10 mice per pen.

### Experiment 3: Protective effects of DMG-Na against LPS-induced oxidative stress in mice

**Fig 4:**
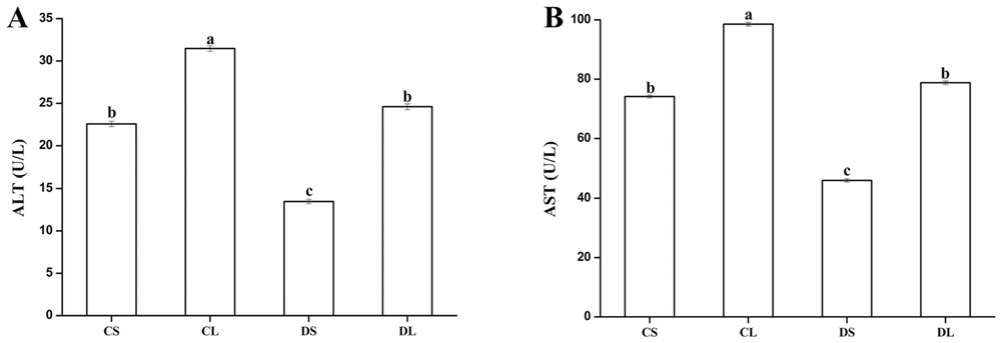
Effects of DMG-Na on ALT (A) and AST (B) activities in LPS-injected mice serum. Values are mean ± SEMs (n = 10). Mean of a variable without a common letter differ, *P* < 0.05. Alanine aminotransferase (ALT); Aspartate aminotransferase (AST). CS, gastric intubation in mice with 0 mg DMG-Na/0.3 ml sterile saline solution for 28 days and intraperitoneal injection with 100 μg/kg body weight of sterile saline solution at 29 day; CL, gastric intubation in mice with 0 mg DMG-Na/0.3 ml sterile saline solution for 28 days and intraperitoneal injection with 100 μg/kg body weight of LPS at 29 day; DS, gastric intubation in mice with 12 mg DMG-Na/0.3 ml sterile saline solution for 28 days and intraperitoneal injection with 100 μg/kg body weight of sterile saline solution at 29 day; DL, gastric intubation in mice with 12 mg DMG-Na/0.3 ml sterile saline solution for 28 days and intraperitoneal injection with 100 μg/kg body weight of LPS at 29 day.

Mice in the CL group showed a significantly increased content of ALT and AST in the serum (*P* < 0.05) in response to LPS exposure, as compared to the CS group (Fig 4A and 4B). The mice in the DL group had significantly lower ALT and AST contents in the serum compared to that observed for mice in the CL group (*P* < 0.05).

**Fig 5:**
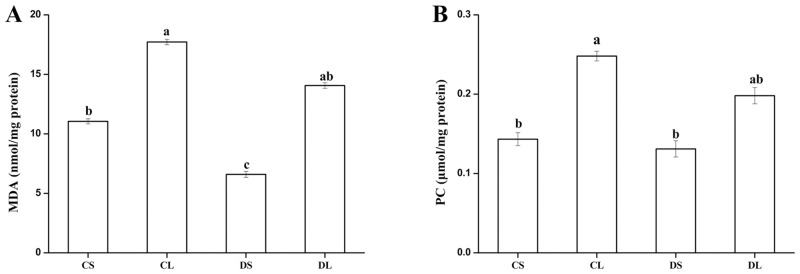
Effects of DMG-Na on mitochondrial MDA (A) and PC (B) concentrations in LPS-injected mice liver. Values are mean ± SEMs (n = 10). Mean of a variable without a common letter differ, *P* < 0.05. Malondialdehyde (MDA); Protein carbonyl. (PC). CS, gastric intubation in mice with 0 mg DMG-Na/0.3 ml sterile saline solution for 28 days and intraperitoneal injection with 100 μg/kg body weight of sterile saline solution at 29 day; CL, gastric intubation in mice with 0 mg DMG-Na/0.3 ml sterile saline solution for 28 days and intraperitoneal injection with 100 μg/kg body weight of LPS at 29 day; DS, gastric intubation in mice with 12 mg DMG-Na/0.3 ml sterile saline solution for 28 days and intraperitoneal injection with 100 μg/kg body weight of sterile saline solution at 29 day; DL, gastric intubation in mice with 12 mg DMG-Na/0.3 ml sterile saline solution for 28 days and intraperitoneal injection with 100 μg/kg body weight of LPS at 29 day.

Compared to the CS group, in the CL group, the activities of the mitochondrial antioxidant system significantly decreased (*P* < 0.05, Table 4), and MDA and PC contents significantly increased (*P* < 0.05) in response to LPS injection (Fig 5A and 5B). In comparison to the mice of the CL group, those in the DL group had significantly higher activities of the mitochondrial antioxidant system (*P* < 0.05), but had lower (*P* > 0.05) MDA and PC contents in the liver mitochondrial.

**Table 4:** Effects of DMG-Na on mitochondrial antioxidant system in LPS-injected mice liver ^1^.

	Treatment ^3^			
Item ^2^	CS	CL	DS	DL
**MnSOD (U/mg protein)**	98.04±1.25^b^	41.05±1.07^d^	112.18±2.31^a^	78.53±1.20^c^
**GSH (U/mg protein)**	0.44±0.03^b^	0.25±0.02^c^	0.71±0.04^a^	0.38±0.02^b^
**GPx (U/mg protein)**	49.60±0.19^b^	21.35±0.14^d^	55.17±0.25^a^	28.06±0.25^c^
**GR (U/mg protein)**	2.91±0.09^b^	0.73±0.02^d^	3.18±0.05^a^	1.36±0.06^c^

^a-d^ Means in the same row with different superscripts differ (*P* < 0.05).

^1^ CS, gastric intubation in mice with 0 mg DMG-Na/0.3 ml sterile saline solution for 28 days and intraperitoneal injection with 100 μg/kg body weight of sterile saline solution at 29 day; CL, gastric intubation in mice with 0 mg DMG-Na/0.3 ml sterile saline solution for 28 days and intraperitoneal injection with 100 μg/kg body weight of LPS at 29 day; DS, gastric intubation in mice with 12 mg DMG-Na/0.3 ml sterile saline solution for 28 days and intraperitoneal injection with 100 μg/kg body weight of sterile saline solution at 29 day; DL, gastric intubation in mice with 12 mg DMG-Na/0.3 ml sterile saline solution for 28 days and intraperitoneal injection with 100 μg/kg body weight of LPS at 29 day.

^2^ Manganese superoxide dismutase (MnSOD), Glutathione (GSH), Glutathione peroxidase (GPx), Glutathione reductase (GR).

^3^ Each mean represents one replicate pens with 10 mice per pen.

**Fig 6:**
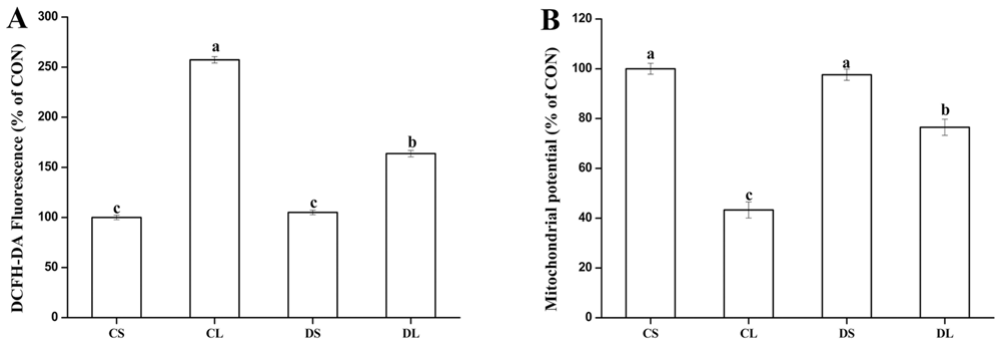
Effects of DMG-Na on ROS concentrations (A) and mitochondrial membrane potential (B) in LPS-injected mice liver. In panels A and B, the value of CS group is set to 100%. Values are mean ± SEMs (n = 10). Mean of a variable without a common letter differ, *P* < 0.05. Reactive oxygen species (ROS). CS, gastric intubation in mice with 0 mg DMG-Na/0.3 ml sterile saline solution for 28 days and intraperitoneal injection with 100 μg/kg body weight of sterile saline solution at 29 day; CL, gastric intubation in mice with 0 mg DMG-Na/0.3 ml sterile saline solution for 28 days and intraperitoneal injection with 100 μg/kg body weight of LPS at 29 day; DS, gastric intubation in mice with 12 mg DMG-Na/0.3 ml sterile saline solution for 28 days and intraperitoneal injection with 100 μg/kg body weight of sterile saline solution at 29 day; DL, gastric intubation in mice with 12 mg DMG-Na/0.3 ml sterile saline solution for 28 days and intraperitoneal injection with 100 μg/kg body weight of LPS at 29 day.

There was a significant increase (*P* < 0.05) in ROS concentration and a significant decrease (*P* < 0.05) in MMP level in the CL group mice compared to that observed for the mice in the CS group (Fig 6A and 6B). Compared to the CL group, the DL group exhibited significantly decreased ROS concentration (*P* < 0.05) and significantly increased MMP level (*P* < 0.05) (Fig 6A and 6B).

**Fig 7:**
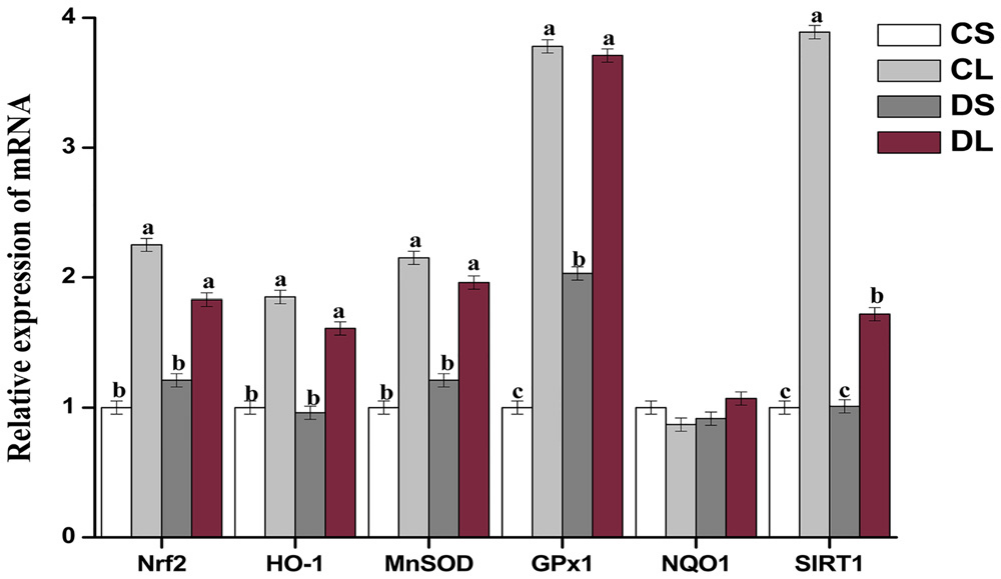
Effects of DMG-Na on mRNA expression of antioxidant genes in LPS injected mice liver. Values are mean ± SEMs (n = 10). Mean of a variable without a common letter differ, *P* < 0.05. Nuclear factor erythroid 2-related factor 2 (*Nrf2*); Heme oxygenase 1 (*HO-1*); Manganese superoxide dismutase (*MnSOD*); Glutathione peroxidase 1 (*Gpx1*); NAD(P)H quinone oxidoreductase 1 (*NQO1*); Sirtuin 1 (*Sirt1*). CS, gastric intubation in mice with 0 mg DMG-Na/0.3 ml sterile saline solution for 28 days and intraperitoneal injection with 100 μg/kg body weight of sterile saline solution at 29 day; CL, gastric intubation in mice with 0 mg DMG-Na/0.3 ml sterile saline solution for 28 days and intraperitoneal injection with 100 μg/kg body weight of LPS at 29 day; DS, gastric intubation in mice with 12 mg DMG-Na/0.3 ml sterile saline solution for 28 days and intraperitoneal injection with 100 μg/kg body weight of sterile saline solution at 29 day; DL, gastric intubation in mice with 12 mg DMG-Na/0.3 ml sterile saline solution for 28 days and intraperitoneal injection with 100 μg/kg body weight of LPS at 29 day.

Mice in the CL group injected with LPS showed an increase (*P* < 0.05) in the mRNA levels of *Nrf2*, *HO-1*, *MnSOD*, *Gpx1*, and *Sirt1* in their liver when compared to those in the CS group (Fig 7). The mRNA levels of *MnSOD*, *Gpx1*, and *Sirt1* were significantly lower (*P* < 0.05) in the DL group than in the CL group.

## Discussion

Trolox, a water-soluble analog of vitamin E, is a powerful free radical scavenger and possesses good antioxidant activity. It has been found to be potent in providing protection against oxyradical induced hepatic injury. The rapid and efficient antioxidant property of Trolox is attributed to its water solubility [27]. Assay of DPPH radical scavenging activity, one of the standard and easy colorimetric methods, is widely used for its simplicity and reproducibility [28]. DPPH is a stable nitrogen radical that has been widely used to measure the ability of antioxidants to inhibit pre-formed free radicals [29]. Addition of reducers to a DPPH solution induces a rapid change of color, which indicates the formation of a stable diamagnetic molecule [30]. Antioxidant reaction with DPPH could neutralize the excessive free radicals by transfer of either an electron or a hydrogen atom to DPPH [31]. Assay of radical scavenging activities might be influenced by many factors. Hence, another *in vitro* assay, such as the ABTS^+^ radical scavenging activity measurement, should be considered. ABTS^+^ is a peroxidase substrate for both lipophilic and hydrophilic antioxidants, and produces a metastable radical with a blue-green color upon oxidation in the presence of H_2_O_2_ [32]. ABTS^+^ decolorization assay is used for the rapid measurement of the total antioxidant activity of individual chemical compounds [33]. In the present study, DMG-Na (40 mg/ml) showed the highest DPPH and ABTS^+^ radical scavenging activity compared with Trolox, indicating the potential of DMG-Na as a free radical scavenger. DPPH and ABTS^+^ radicals, with high reproducibility and excellent stability, are the two most widely used chromogenic compounds to calculate the capacity of free radical inhibition. Although values of DPPH and ABTS^+^ free radical inhibition corresponded with each other, our results suggest that DMG-Na reacted more actively with the ABTS^+^ solution than with DPPH solution.

Hydroxyl radicals, the most reactive of all reduced forms of dioxygen studied in recent years, along with other ROS act in the initiation of cell damage and lipid peroxidation, *in vivo* [34]. H_2_O_2_ is relatively stable compared to other ROS and is the only ROS that diffuses through the aquaporin proteins present in the cell membranes [35]. H_2_O_2_ also plays a dual role *in vivo* by acting as a signaling molecule at low concentrations [36], but causes lipid peroxidation, cellular destruction, protein denaturation, and DNA damage at higher concentrations [37]. Removal of excess H_2_O_2_ is crucial in the antioxidant defense of an individual. In the present study, DMG-Na (40 mg/ml) showed 87.58% of the H_2_O_2_ scavenging activity of Trolox, and the results were highly similar to the scavenging activity of other radicals, such as DPPH and ABTS^+^. This suggests that DMG-Na has the potential of neutralizing free radicals, *in vitro*. The current results were supported by studies of Hariganesh and Prathiba [19], who suggested the free-radical scavenging potential of DMG during oxidative stress in rats after oral administration. However, little is known about the exact effect and mechanism of free radical scavenging activity of DMG-Na.

DMG could promote the synthesis of proteins, enhance the ability of protection against oxidative stress in animals, and improve their health condition [38]. The development of internal organs is related to physiological functions. The organ index is an important indicator of the metabolic functions of the body. Size of immune organs is one of crucial indicators reflecting the immune capacity of animals, smaller the immune organ, weaker is the immunity [39]. In the present study, DMG-Na was beneficial to the growth performance and organ proportion in mice. In agreement with our results, some studies have been suggested that DMG-Na could improve the growth performance of broilers [40,41], for the emulsifying action of DMG-Na at the intestinal tract [41]. DMG-Na also acts as a source of glycine for glutathione synthesis [11,12] and leads to the improvement of protein biosynthesis [17]. However, uncertainty exists about the mechanism through which DMG-Na improves the growth performance and organ proportion in mice.

The increasing level of ALT and AST, considered as toxicity markers, could be due to hepatic injury [42]. ALT plays an important role in metabolism of amino acids, which usually relates to cell damage and rupture. AST is present in mitochondria and cytoplasm. Similar to our results, treatment with radical scavenger of EGCG effectively reduced CCl4-induced oxidative stress and diminished the serum ALT level [43]. Hsu et al. [44] also suggested that treatment of sesame oil-treated groups with an anti-lipid peroxidation molecule attenuated hepatic injury, and reduced serum AST level induced by LPS. *T*. *officinale*, as a radical scavenger, could improve levels of serum ALT and AST induced by oxidative stress [45]. The present study shows that the administration of DMG-Na effectively attenuated the progression of LPS-induced oxidative stress in mice.

Lipid peroxidation, which normally occurs at lower levels in an individual, is a common product of oxidative stress. However, excess ROS in cells may result in cell and tissue damage. During their growth, animals produce free radicals in their body through enzymatic and non-enzymatic systems, resulting in oxidative stress and reduction of antioxidant ability. Antioxidant enzymes, including SOD, CAT, and GPx, could help avoid these damages induced by oxidation, and play an important role in decreasing the levels of ROS [46]. The antioxidant defense system inhibits the ROS, which are a major cause of oxidative stress. Antioxidant enzymes appear to be the first line of defense during ROS inhibition process. SOD promotes the production of O_2_ and H_2_O_2_ from O_2_^-^, which in turn are decomposed to water by CAT and GPx enzymes, thus avoiding the formation of OH. Catalase is one of the crucial antioxidant enzymes that protect the body from oxidative stress caused by ROS [47]. Enzyme activity of CAT is usually affected by superoxide radicals [48]. GPx is one of the most important antioxidant enzymes that act against free radicals and lipid peroxidation [49]. T-AOC plays a crucial role in the antioxidant defense system of an individual. It inhibits the ROS and prevents lipid peroxidation by blocking the peroxidation chain. Similar to our results, Hariganesh et al [19]. reported that gastric intubation in mice with DMG could eliminate free radicals and reduce tissue injury caused by oxidative stress. DMG could have enhanced the antioxidant capacity of animals, through its own electronic chelate ligand passivation of pro-oxidant metal ions, which might be one of the reasons for improved total antioxidant capacity by DMG-Na in animals [50]. Mitochondrial antioxidant activity in LPS-challenged mice liver was increased by DMG-Na. One possible mechanism leading to oxidative stress is the disruption of redox status [51]. The ROS defense system in the mitochondria include enzymatic (MnSOD, GPx, and GR) and nonenzymatic (mainly GSH) antioxidants. Hariganesh et al. [19] suggested antioxidant capacity of DMG by increasing the number of antioxidant enzymes, and significant improvement against oxidative stress when orally administered to rats. Kalmar et al. [18] also reported that DMG has anti-oxidative properties and could improve the oxidative stress in broilers. The above results indicate that DMG-Na could enhance the ability of the antioxidant defense systems and eliminate excess of free radicals.

Mitochondria are one of the most vulnerable targets of free radicals for the enrichment of PUFAs (polyunsaturated fatty acids) in their membranes, and produce more than 95% of ROS in the body. ROS exert different effects depending on their cellular concentration; moderate ROS level improves cell proliferation, development, and differentiation, whereas excess production usually results in oxidative stress and inhibits the normal functioning of cellular proteins, DNA, and RNA [52]. The body can produce oxygen free radicals causing destruction of the PUFAs present in the membranes, and leads to the enhancement of lipid peroxide’s ability to cause oxidative stress [53]. Under oxidative stress, excess free radicals interact with mitochondrial membranes, causing potential damage and increase of MDA contents [54]. Consistent with other results [55–57], we also showed that LPS increased ROS contents, and decreased MMP in mitochondria of mice liver. One possible explanation of the above results is that DMG-Na, as an antioxidant, could scavenge excess free radicals efficiently against oxidative stress in mitochondrial membranes. Studies also reported on the ability of antioxidants in directly preventing lipid peroxidation via its strong free radical scavenging capacity [58]. MDA is one of the most important final products of peroxidation of unsaturated fatty acids in phospholipids, and cause damage to the cell membranes. The concentrations of MDA could indirectly reflect the extent of an individual’s oxidative stress. Our results were consistent with that of Hariganesh, who indicated that mice gastric intubation with DMG could significantly reduce the MDA contents in blood and tissues [19]. The protein carbonyls, another indicator of oxidative stress, are produced through the modification of protein molecules by oxidation [59]. We also showed that LPS injection could significantly increase the concentrations of ROS, MDA, and PC in the mitochondria of mice liver. Treatment with DMG-Na could improve the oxidative stress through decreasing concentrations of mitochondrial MDA and ROS in the LPS injected mice. Similar to our results, Colle et al. [45] found that *T*. *officinale*, as a radical scavenger, could protect body from oxidative stress by removing excess of free radicals. Hariganesh et al. [19] suggested that DMG had antioxidation potential by reducing the level of reactive thiobarbituric acid when orally administered to rats. Brun et al. [60] also suggested that LPS injection might contribute to oxidative stress by generating ROS in mitochondria, while the anti-oxidant therapy may be helpful in the treatment of oxidative stress. One explanation for the above results is that DMG-Na used as an antioxidant could scavenge the excess of free radicals and thereby offer protection against oxidative stress induced by LPS.

DMG-Na, acting as a methyl donor, could improve body immunity, function as an antioxidant, prevent oxidative stress, and scavenge excess free radicals to avoid unwanted reactions in the body [19]. The current results were similar to those reported by Zhang et al. [56], Clerch and Massaro [61], and Virgili et al. [62]. As we know, mRNA could be translated to functional proteins in the ribosome, endoplasmic reticulum, and Golgi of normal cells. These organelles could be damaged by ROS produced by LPS injection [6,8], thereby decreasing the protein translational efficiency and their effectiveness [63]. In agreement with our results, Lambertucci et al. [64] suggested that the increase in the expression of antioxidant enzyme genes were due to the oxidative stress in body, and the expression of antioxidant enzyme genes in DMG-Na-treated groups were lower than that in the LPS-treated groups, for DMG-Na could suppress oxidative stress by inhibiting the ROS produced by LPS-injection [19]. One possible explanation for the decreasing activities of antioxidant enzyme proteins in LPS-treated mice is the degraded oxidative stress induced by LPS [65]. *Sirt1* improves the oxidative stress in cells through the induction of Forkhead box O 3 (*Foxo 3*), which could directly affect the expression of some antioxidant genes [66]. Previous study also verified the protective effects of a radical scavenger against oxidative stress via the regulation of *Sirt1* [67]. It would be worthwhile to do further study on the effect of DMG-Na on oxidative stress.

In conclusion, the current study indicated that DMG-Na may prove to be a potential antioxidant, and could ameliorate the oxidative stress present in LPS-induced mice. This could be due to the presence of high radical scavenging activity and enhancement of the endogenous antioxidant defense system, as well as improvement in the oxidative stress induced gene expression. In future, we hope that more attention will be given to elucidate the molecular mechanism of DMG-Na in the LPS model.
